# Opportunistic use of chest CT for screening osteoporosis and predicting the risk of incidental fracture in breast cancer patients: A retrospective longitudinal study

**DOI:** 10.1371/journal.pone.0240084

**Published:** 2020-10-14

**Authors:** So Hyun Park, Yu Mi Jeong, Hee Young Lee, Eun Young Kim, Jeong Ho Kim, Heung Kyu Park, Hee Kyung Ahn

**Affiliations:** 1 Department of Radiology, Gil Medical Center, Gachon University College of Medicine, Namdong-gu, Incheon, Korea; 2 Department of Surgery, Breast Cancer Center, Gil Medical Center, Gachon University College of Medicine, Incheon, Korea; 3 Department of Oncology, Department of Internal Medicine, Gil Medical Center, Gachon University College of Medicine, Incheon, Korea; Medical College of Wisconsin, UNITED STATES

## Abstract

This study aimed to investigate the diagnostic performance of chest computed tomography (CT) for opportunistic screening and longitudinal follow-up of osteoporosis in breast cancer patients, compared to dual-energy X-ray absorptiometry (DXA). The association between L1 vertebral attenuation on chest CT and incidental fracture was also evaluated. We retrospectively reviewed 414 consecutive breast cancer patients who underwent both non-enhanced chest CT and DXA within a 3-month interval and had at least two DXA and two chest CT examinations over more than 1 year. The attenuation value of the L1 trabecular bone was measured on an axial CT image and compared to the corresponding DXA T-score. The diagnostic performance of L1 vertebral attenuation on CT for osteoporosis was calculated at different thresholds (90 HU, 100 HU, 110 HU), and the correlation between L1 vertebral attenuation values and DXA T-scores was statistically analyzed. Overall fracture-free survival was estimated and compared with the threshold of 90 HU on CT and -2.5 T-score on DXA. Of 414 patients (median age, 53.0 years), 88 (21.3%) had either vertebral or non-vertebral fractures. The median follow-up duration between initial and final DXA was 902.9 days. There was a moderate correlation between L1 vertebral attenuation value and DXA T-score (ρ = 0.684, CI 0.653–0.712). Fracture-free survival was significantly lower in patients with attenuation values ≤90 HU on CT and T-scores ≤-2.5 on DXA (*P* < .001). Multivariate analysis revealed that attenuation values ≤90 HU on CT (*P* < .001), T-scores ≤-2.5 on DXA (*P* = .003), and age ≥65 years (*P* = .03) were independent significant prognostic factors associated with overall fracture-free survival. The sensitivities and specificities of L1 attenuation value were 54.9% and 85.8% at 90-HU threshold, 74.0% and78.4% at 100-HU threshold, and 83.9% and 70.1% at 110-HU threshold, respectively. In conclusion, CT can be used for predicting osteoporosis and discriminating incidental fracture risk in breast cancer patients.

## Introduction

The current National Comprehensive Cancer Network (NCCN) guidelines in the United States recommend that women with breast cancer who have high-risk factors for osteoporosis, including those with a family history of fractures, body weight less than 70 kg, prior non-traumatic fractures, postmenopausal and receiving aromatase inhibitor therapy, and premenopausal with therapy-induced ovarian failure, should undergo bone mineral density (BMD) assessment and regular monitoring [1,2]. In addition, the NCCN Guidelines propose that baseline and periodic follow-up BMD should be assessed in patients with breast cancer who receive sex steroids for bone health and fracture risk assessment [2].

Currently, dual-energy X-ray absorptiometry (DXA) is the gold-standard method for non-invasive BMD assessment due to its intermediate cost, low radiation exposure, excellent precision, and ability to monitor treatment response [2]. However, the variability among technicians in operating DXA equipment, the use of different settings for the dual-energy methods, and the presence of vertebral compression fractures, osteoarthritis, osteomalacia, or aorta calcification in patients can affect the results of BMD measurement [2,3]. There are several limitations of BMD associated with the patient, technician, machine, and clinician [4–6].

Chest CT is an imaging modality widely used in breast cancer patients to evaluate pulmonary symptoms or cancer spread. Regardless of the cancer stage, many clinical institutions currently perform chest CT in patients with breast cancer as a baseline and follow-up imaging modality [7]. More recently, feasibility studies have supported that CT measurement of vertebral attenuation during abdomen or chest CT performed for other indications can be used for opportunistic osteoporosis screening or evaluation of bone density loss [8–10]. Moreover, vertebral attenuation could be used to predict future osteoporotic fracture risk [11]. Potentially, vertebral attenuation on chest CT scans may show a similar performance to DXA in diagnosing osteoporosis and discriminating risk for incidental fracture in breast cancer patients. In such a scenario, measurement of vertebral attenuation on chest CT could reduce the need for DXA in BMD evaluation and help predict bone loss in breast cancer patients. However, to our knowledge, this application of the imaging technique has not yet been explored.

Therefore, this study evaluated the performance of chest CT in diagnosing osteoporosis and following up patients compared to that of DXA, as well as the association between L1 vertebral attenuation on chest CT and incidental fracture in breast cancer patients.

## Materials and methods

This retrospective study was approved by the Institutional Review Board, Gachon University Gil Medical Center (GDIRB2020-173). The requirement for obtaining written informed patient consent was waived because the study involved no more than minimal risk and the waiver would not adversely affect the rights and welfare of the subjects.

### Study population

**Fig 1:**
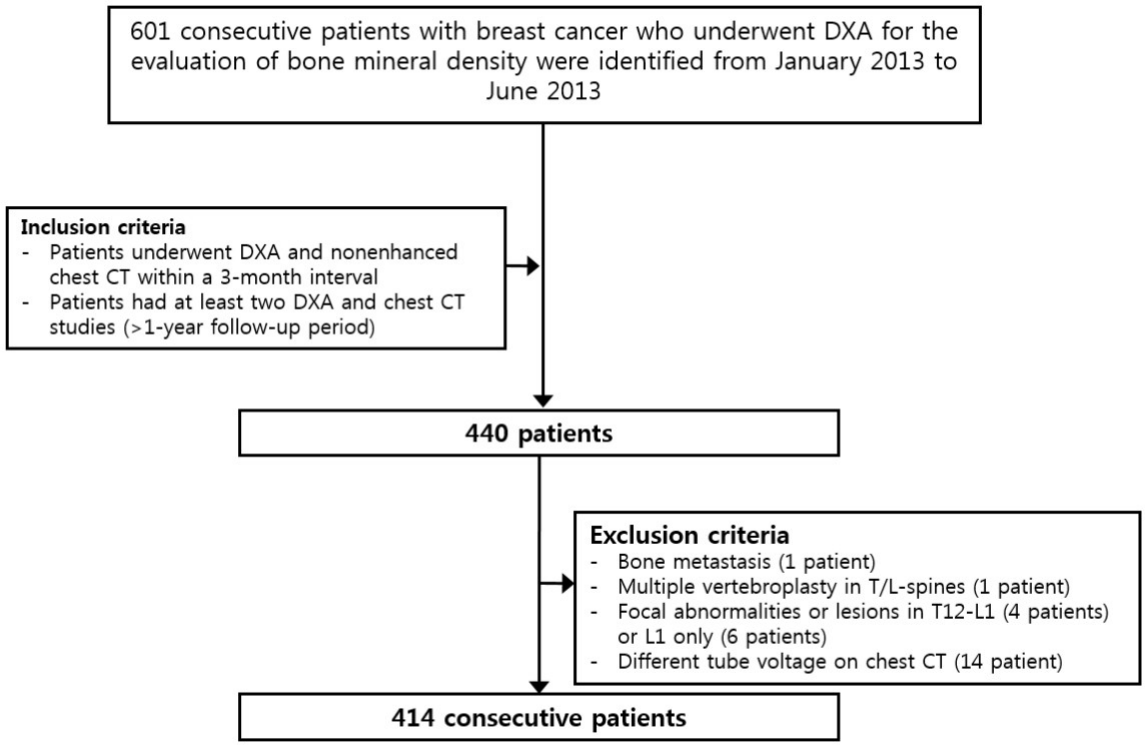
Patient’s flowchart.

In total, 601 consecutive patients with breast cancer who underwent DXA for the evaluation of iBMD were identified at Gachon University Gil Medical Center (Incheon, Korea) from January 2013 to June 2013. Of them, all patients who underwent DXA and non-enhanced chest CT within a 3-month interval and had at least two DXA and two chest CT studies over a follow-up period of more than 1 year were included. As a result, 440 patients were identified. We further excluded 1 patient with bone metastases in multiple vertebral bodies, 1 patient with a multiple vertebroplasty in T/L spines, and 4 patients with focal abnormalities or lesions in T12-L1 for whom a reliable L1 trabecular measurement was not feasible. Six patients were excluded because of prior L1 compression fractures. Another 14 patients were excluded due to the use of different tube voltages at different times during the follow-up (i.e. 100 kVp and 120 kVp). Thus, a total of 414 consecutive patients were included in the study (Fig 1). The medical records of all enrolled patients were reviewed. Potential covariates of incident fracture during the study period, including age at initial CT, sex, body mass index, smoking history, alcoholism, glucocorticoid use, rheumatoid arthritis history, hypertension, diabetes mellitus, aromatase inhibitor use, chemotherapy, and vitamin D therapy were also collected.

### DXA acquisition technique

Central DXA (Lunar Prodigy Advance, GE Healthcare, Waukesha, Wisconsin) of the L-spine and proximal femur was performed for BMD assessment using standard techniques according to the 2019 International Society for Clinical Densitometry official positions [12]. The measured BMD was converted into the T-score using the manufacturer-provided Korean (aged 20–40) reference population. For inclusion, at least one valid T-score report for the lumbar spine or hips was required. Osteoporosis was diagnosed in breast cancer patients when the DXA T-score of the lumbar spine, total hip, or femoral neck was -2.5 or less. Osteopenia was defined as a DXA T-score of between -1.0 and -2.4. The lowest T-score at one of the 3 skeletal sites was chosen for the diagnosis of osteopenia or osteoporosis. [12,13]. A reviewer (YMJ) who was blinded to the results of CT attenuation interpreted the DXA results.

### CT scan technique

Non-enhanced chest CT scans were performed on different multi-detector scanners (SOMATOM Edge, SOMATOM Definition AS, and SOMATOM Definition Flash, Siemens Healthcare) at a constant peak voltage of 120 kV with variable protocol-specific tube current (mA) settings. The kVp settings have a strong effect on bony Hounsfield unit (HU) values. The CT scan with intravenous contrast material also has a measurable effect on bony attenuation values [9]. Image data were acquired in helical mode with a slice thickness of 1 mm, and axial, coronal, and sagittal reformation was done with a slice thickness of 3 mm. The scan range was from thoracic inlet to upper abdomen, so L1-L2 vertebrae were always included in the scan. All CT scanners in our institution underwent routine daily quality assurance calibration to ensure scanner stability.

### Image analysis

A single reviewer (HYL, board-certified, fellowship-trained thoracic radiologist with 9 years of clinical experience), blinded to the DXA results, measured mean L1 trabecular attenuation on a single axial CT image at the appropriate level by manually placing an ovoid region of interest (ROI) within the anterior-superior portion of the trabecular space while avoiding cortical bone and focal sclerotic or lytic lesions [9,14]. In the event of a compression fracture at L1, either T12 or L2 were utilized for trabecular attenuation measurement. The reviewer was blinded to patient DXA as well as to medical treatment while measuring L1 attenuation. The reviewer visually assessed sagittal CT images to determine the presence of vertebral fractures. We applied semiquantitative approach to define vertebral fractures [15]. Vertebral compression fracture was determined when apparent reduction of anterior vertebral height greater than 20% was observed on sagittal CT. The presence of vertebral fractures were confirmed by a board-certified, fellowship-trained musculoskeletal radiologist (YMJ) with 11 years of clinical experience in a separate reading session with reference to other available imaging examinations such as serial spine X-ray, spine MRI, or bone scan. In addition, the reviewer (YMJ) also investigated other imaging examinations of patients to find non-vertebral fractures. Image assessment and measurements were all performed using our institutional picture archiving and communication system (PACS).

### Statistical analysis

Clinical characteristics were compared between patients with and without fractures using Student’s t-test or Mann-Whitney U-test for continuous variables and chi-square test for categorical variables. Continuous variables are given as mean ± standard deviation or median (interquartile range [IQR]), depending on the normality of the distribution. Statistical correlation tests between L1 DXA T-score and L1 vertebral attenuation, L spine DXA T-score and L1 vertebral attenuation, DXA T-scores and L1 vertebral attenuation values, respectively for the initial paired examination relied on Spearman rank correlation coefficient (ρ). Correlation between DXA T-scores and L1 vertebral attenuation values among the entire paired examinations were analyzed using Spearman rank correlation coefficient (ρ) and Deming regression. The diagnostic performance of L1 vertebral attenuation on chest CT based on the thresholds of 90 HU (≥90 vs. <90 HU), 100 HU (≥100 vs. <100 HU), and 110 HU (≥110 vs. <110 HU) were measured in terms of sensitivity and specificity with 95% confidence intervals (CIs) for DXA-diagnosed osteoporosis cases. Overall fracture-free survival (excluding preexisting fractures) was estimated using Kaplan–Meier survival curves and compared between the L1 CT attenuation > 90 HU and ≤90 HU groups and between the DXA T-score >-2.5 and ≤-2.5 groups using the log-rank test. The Cox proportional hazard model was used for univariate and multivariate analysis of factors related to overall fracture-free survival. Variables with a *P* < .1 via univariate analyses were included in multivariate survival analysis, and *P* < .05 was considered significant. Statistical analyses were performed using medcalc and R 3.6.3 (https://www.r-project.org).

## Results

### Patient clinical characteristics

Clinical characteristics of patients are summarized in Table 1. Of the 414 enrolled, all patients were women with a median age (interquartile range) of 53.0 years (49.0–59.0). A total of 326 (78.7%) patients were non-fracture cases and 88 (21.3%) were fracture cases.

**Table 1:** Characteristics of breast cancer patients.

Variables	Total	Non-fracture	Fracture	p
	(N = 414)	(N = 326)	(N = 88)	
Age (years) at initial CT	53.0 (49.0–59.0)	52.0 (48.0–57.0)	56.0 (51.0–62.5)	< 0.001
Height (cm)	157.0 (154.0–160.0)	158.0 (154.0–160.0)	156.5 (153.0–160.0)	0.335
Weight (kg)	58.0 (53.0–64.0)	58.0 (53.0–64.0)	57.0 (54.0–64.0)	0.980
BMI (kg/m^2^)	23.5 (21.6–25.7)	23.4 (21.5–25.8)	23.8 (22.2–25.3)	0.353
BMI_Catgory				0.883
BMI < 18.5	10 (2.4%)	8 (2.5%)	2 (2.3%)	
BMI 18.5–24.9	270 (65.2%)	210 (64.4%)	60 (68.2%)	
BMI 25–29.9	115 (27.8%)	94 (28.8%)	21 (23.9%)	
BMI 30–34.9	16 (3.9%)	12 (3.7%)	4 (4.5%)	
BMI >35	3 (0.7%)	2 (0.6%)	1 (1.1%)	
Smoking				0.775
Nonsmoker	402 (97.1%)	317 (97.2%)	85 (96.6%)	
Former smoker	11 (2.7%)	8 (2.5%)	3 (3.4%)	
Current smoker	1 (0.2%)	1 (0.3%)	0 (0.0%)	
Alcoholism				0.728
No	400 (96.6%)	316 (96.9%)	84 (95.5%)	
Yes	14 (3.4%)	10 (3.1%)	4 (4.5%)	
Rheumatoid arthritis				0.536
No	409 (98.8%)	321 (98.5%)	88 (100.0%)	
Yes	5 (1.2%)	5 (1.5%)	0 (0.0%)	
Glucocorticoids				1.000
No	320 (77.3%)	252 (77.3%)	68 (77.3%)	
Yes	94 (22.7%)	74 (22.7%)	20 (22.7%)	
DM				0.069
No	372 (89.9%)	298 (91.4%)	74 (84.1%)	
Yes	42 (10.1%)	28 (8.6%)	14 (15.9%)	
HTN				0.469
No	311 (75.1%)	248 (76.1%)	63 (71.6%)	
Yes	103 (24.9%)	78 (23.9%)	25 (28.4%)	
Aromatase inhibitor				0.383
No	383 (92.5%)	304 (93.3%)	79 (89.8%)	
Yes	31 (7.5%)	22 (6.7%)	9 (10.2%)	
Chemotherapy				0.629
No	200 (48.3%)	160 (49.1%)	40 (45.5%)	
Yes	214 (51.7%)	166 (50.9%)	48 (54.5%)	
Vit. D therapy				0.526
No	53 (12.8%)	44 (13.5%)	9 (10.2%)	
Yes	361 (87.2%)	282 (86.5%)	79 (89.8%)	
L1 attenuation				
Initial CT (HU)	120.1 (92.0–151.0)	126.0 (100.0–154.0)	91.0 (67.0–130.0)	< 0.001
Last follow-up CT (HU)	110.5 (82.1–138.0)	117.5 (91.0–143.0)	81.0 (62.0–118.0)	< 0.001
DXA				
Initial score	-1.9 (-2.6−-1.1)	-1.8 (-2.4−-1.0)	-2.5 (-3.1−-1.8)	< 0.001
Last follow-up score	-2.1 (-2.6−-1.4)	-1.9 (-2.5−-1.3)	-2.6 (-2.9−-1.8)	< 0.001

Values are presented as number (%), median (interquartile range). BMI, body mass index; CT, computed tomography; HU, Hounsfield unit; DXA, dual-energy X-ray absorptiometry.

* We interpreted DXA results at the lowest T-score at one of the 3 skeletal sites (lumbar spine, total hip, femoral neck).

The distribution of incident fractures by body region is presented in Table 2. Altogether, 39 (9.4%) patients underwent two paired DXA and chest CT studies; 332 (80.2%) patients had three paired studies; 38 (9.2%) patients had four paired studies; and 5 (1.2%) patients had five paired studies. The time interval between DXA and chest CT scan within the same year of clinical observation was a median of 32.0 days (0–82 days). The median follow-up duration between initial and final DXA was 902.9 days (368–2377 days), and the median duration between initial DXA and final image studies (i.e. bone scan, chest CT, spine MRI or x-ray) to evaluate fracture was 2211.8 days (767–2945 days). Of the 414 patients, 89 (21.5%) initially presented with L1 attenuation ≤ 90 HU and 122 (29.5%) patients initially presented with DXA ≤ -2.5.

**Table 2:** Distribution of fractures by body region.

Fracture region	No. of fractures (n = 88)
**Rib**	35 (39.8)
**Spinal compression**	21 (23.9)
**Foot/tibia/fibula/femur**	12 (13.6)
**Pelvis/hip**	10 (11.4)
**Wrist/ulna/radius**	8 (9.1)
**Others**	2 (2.3)

Data are presented as the numbers of patients (%).

### Correlation between DXA and chest CT and diagnostic performance of L1 vertebral attenuation on chest CT for DXA-diagnosed osteoporosis

**Fig 2:**
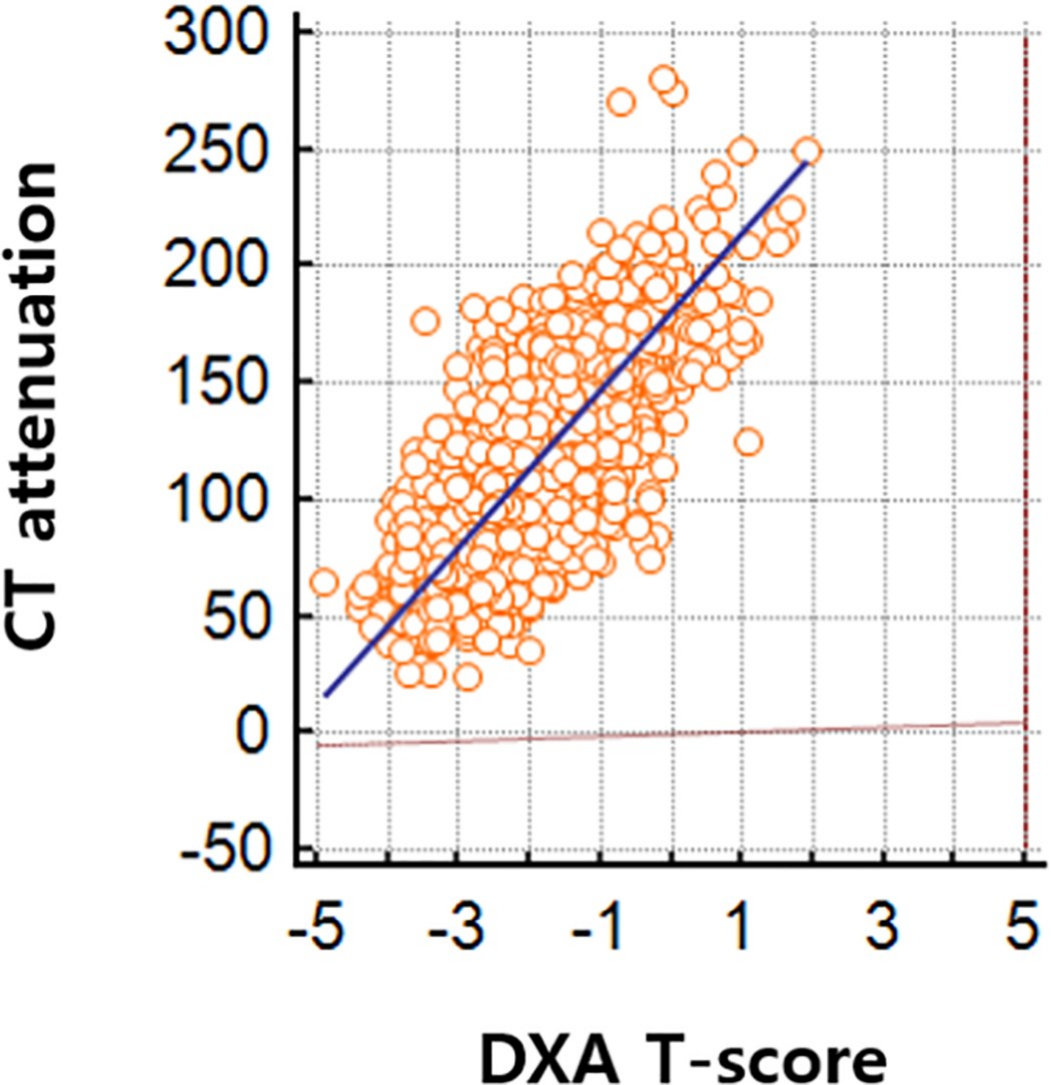
Deming regression between CT attenuation at L1 vertebral body and DXA T-score.

We found that Spearman's coefficient of rank correlation (ρ) among the initial 414 paired examinations was 0.653 (CI, 0.594–0.703) between L1 DXA T-score and L1 vertebral attenuation, 0.612 (CI, 0.548–0.669) between L spine DXA T-score and L1 vertebral attenuation, and 0.693 (CI, 0.639–0.740) between DXA T-score and L1 vertebral attenuation. DXA T-score showed the highest rank correlation with L1 vertebral attenuation among the three measurement of DXA. Correlation between L1 vertebral attenuation and DXA T-score among the 1251 paired studies revealed 0.684 (CI, 0.653–0.712) on Spearman's coefficient and formula using Deming regression; L1 vertebral attenuation = 33.7 × DXA T-score + 181.1 (Fig 2). The diagnostic performances of the three different CT attenuation threshold for osteoporosis are summarized in Table 3. At the 90 HU threshold, the sensitivity was 54.9% and the specificity was 85.8%. At the 100 HU threshold, the sensitivity was 74.0% and the specificity was 78.4%. At the 110 HU threshold, the sensitivity was 83.9% and the specificity was 70.1%.

**Table 3:** Diagnostic performance of L1 vertebral attenuation on chest CT for osteoporosis defined as DXA T-score ≤-2.5.

	Threshold of L1 vertebral attenuation of chest CT		
Diagnostic performance	90 HU	100 HU	110 HU
**Sensitivity**	54.9 (211/384)	74.0 (284/384)	83.9 (322/384)
	[49.8–60.0]	[69.3–78.3]	[79.8–87.3]
**Specificity**	85.8 (744/867)	78.4 (680/867)	70.1 (614/867)
	[83.6–88.3]	[75.8–81.4]	[66.8–73.0]
**Accuracy**	76.3 (955/1251)	77.2 (964/1251)	74.3 (936/1251)
	[74.1–78.8]	[74.8–79.5]	[71.7–76.7]

Data between parentheses are numerator/denominator; data in brackets are 95% confidence interval. AUROC = area under the receiver operating characteristic curve.

### Fracture-free survival according to CT attenuation and DXA score

**Fig 3:**
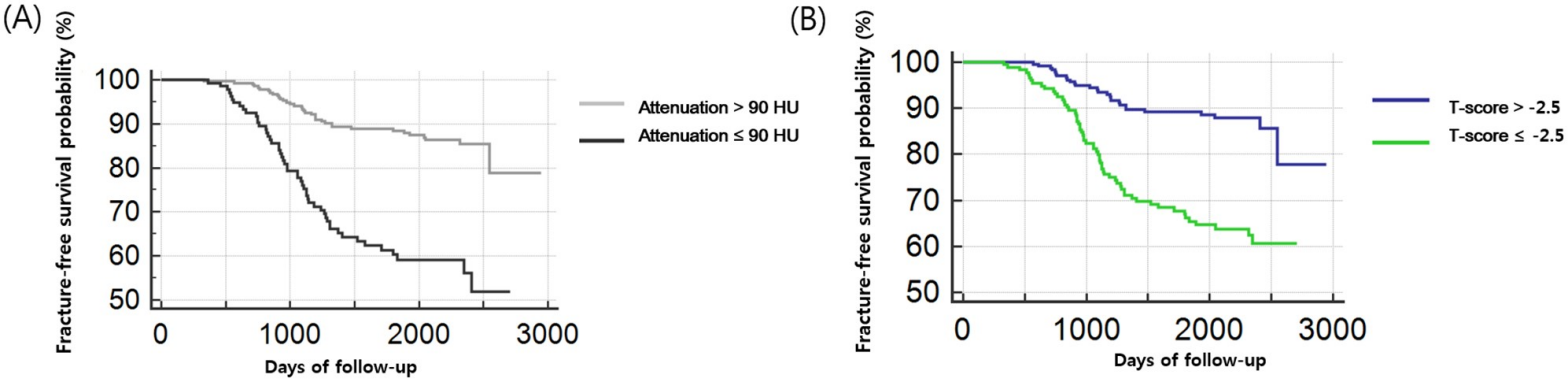
Kaplan–Meier survival curves for fracture-free survival in patients with breast cancer. (A) For comparison, patients were divided into two groups based on the L1 CT attenuation at a 90 HU threshold. Fracture-free survival curves were estimated for each group (L1 attenuation > 90 HU, gray line; L1 attenuation ≤ 90 HU, black line). There was a significant difference in fracture-free survival between the two groups (*P* < .001). (B) Patients were divided into two groups based on the T-score obtained from dual-energy X-ray absorptiometry (DXA) at a -2.5 threshold for comparison. Fracture-free survival curves were estimated for each group (T-score >-2.5, blue line; T-score ≤-2.5, green line). There was a significant difference in fracture-free survival between the two groups (*P* < .001).

The median follow-up duration between the initial DXA and final image studies (i.e. bone scan, chest CT, spine MRI, or x-ray) to evaluate the fracture was 2211.8 days (767–2945 days). The difference in fracture-free survival according to an L1 attenuation threshold of 90 HU (Fig 3A) and according to a threshold of -2.5 T-score on DXA (Fig 3B) are shown in Kaplan-Meier curves. There was a significant difference in fracture-free survival in patients with a threshold of 90 HU of L1 attenuation and -2.5 T-score on DXA (*P* < .001 by log-rank test, respectively).

**Fig 4:**
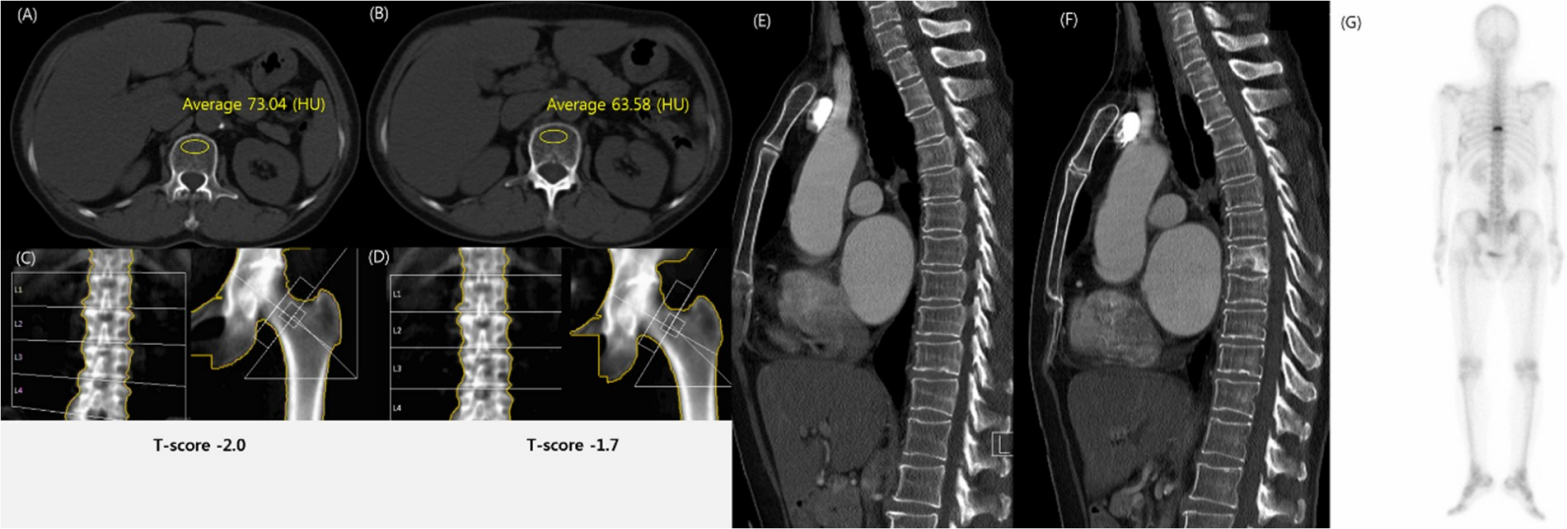
Compression fracture in T8 vertebral body in a 61-year-old woman with breast cancer. (A-B) Chest computed tomography (CT) scan showed a markedly decreased L1 trabecular attenuation in 2013 (73 HU, A) and 2014 (64 HU, B). (C-D) Dual-energy X-ray absorptiometry (DXA) was interpreted as osteopenia with a T-score of -2.0 in 2013 (C) and a T-score of -1.7 in 2014 (D). (E-G) Chest CT scan showed no fracture in 2013 (E). Sagittal CT scan (F) in 2014 and bone scan (G) showed a compression fracture in the T8 vertebral body. The diagnosis discrepancy between DXA and CT in this case suggests that DXA was falsely negative given the subsequently-identified fracture.

In the univariate analysis, incidental fracture-free survival was significantly affected by the ≤90 HU threshold on CT (*P* < .001 for ≤90 HU group, compared with >90 HU group), ≤-2.5 T-score on DXA (*P* = .003 for ≤-2.5 group, compared with >-2.5 group), and age (*P* = .03 for ≥65-year-old group, compared with the 20 to 65-year-old group). Multivariate analysis revealed that belonging to the ≤90 HU group on CT (hazard ratio [HR] = 2.36; *P* < .001), ≤-2.5 group on DXA (HR = 2.13; *P* = .003), and ≥65-year-old at initial CT (HR = 1.80; *P* = .03) were significant independent factors associated with overall fracture-free survival (Table 4, Fig 4, S1a–S1c Fig).

**Table 4:** Cox proportional hazards model for fracture-free survival in patients with breast cancer.

	Univariate analysis		Multivariate analysis	
Variables	Hazard ratio (95% CI)	*P* Value	Hazard ratio (95% CI)	*P-*value
**Age (years)**				
**20–65 y**	1		1	
**≥65 y**	1.91 (1.10–3.32)	.02	1.80 (1.06–3.06)	.03
**Hypertension**	0.90 (0.50–1.5)	.53		
**Diabetes Mellitus**	1.43 (0.75–2.77)	.28		
**Alcohol**	0.71 (0.23–2.18)	.55		
**Smoking**	2.09 (0.58–7.56)	.26		
**Steroid**	1.02 (0.61–1.72)	.26		
**CT attenuation**				
**>90 HU**	1		1	
**≤90 HU**	2.21 (1.32–3.67)	.003	2.36 (1.44–3.86)	< .001
**DXA T-score**				
**>-2.5**	1		1	
**≤-2.5**	2.21 (1.34–3.67)	.002	2.13 (1.29–3.51)	.003
**Aromatase inhibitor**	1.33 (0.63–2.81)	.45		
**Body weight**				
**<70 kg**	1	.76		
**≥70 kg**	0.90 (0.41–1.99)	.81		

Data are presented as the number of patients (95% confidence intervals [CI]).

CT, computed tomography; HU, Hounsfield unit; DXA, dual-energy X-ray absorptiometry.

## Discussion

We evaluated osteoporosis using L1 vertebral attenuation in patients with breast cancer undergoing chest CT and found that L1 vertebral attenuation was associated with fracture-free survival. Our study demonstrated that L1 vertebral attenuation data allowed the prediction of osteoporosis using DXA-T-score.

Previous studies reported that the L1 trabecular attenuation cut-off of 90 HU showed significant differentiation of fracture-free survival factors in patients 65 years and older [11,16] using a 120 kVp CT setting and without contrast material injection. In our multivariate analyses, the most significant independent factors influencing fracture-free survival in patients with breast cancer were L1 vertebral attenuation (≤90 HU group); diagnosis of osteoporosis in DXA (≤-2.5 group); and age ≥65 years. Therefore, L1 attenuation values from chest CT may be used for discriminating risk for incidental fracture in patients with breast cancer.

Postmenopausal women with breast cancer frequently experience cancer treatment-induced bone loss [2,17]. Cody et al. suggested that younger breast cancer survivors are also at higher risk of osteoporosis compared to cancer-free women [18]. Breast cancer survivors can be affected by bone loss and have increased risk of fractures later in life. Patients should be made aware of the risk to reduce incidental fracture and improve treatment of bone loss. In the Kaplan–Meier survival curves, L1 CT attenuation ≤90 HU and ≤-2.5 T-score were associated with poor fracture-free survival. Our results provided additional evidence that the bone mineral density of the L1 trabecular attenuation on CT could help detect not only lung nodules but also bone mineral loss in patients with breast cancer. Therefore, chest CT will increase opportunities for identifying risk for incidental fractures and for planning osteoporosis treatment.

Several studies found degenerative changes in the lumbar spine may result in increasing spinal BMD, leading to misinterpretation of BMD in elderly women [19–21]; those findings are consistent with the highest correlation between DXA T-scores and L1 vertebral attenuation values, whereas correlation between L spine DXA T-score and L1 vertebral attenuation revealed the lowest correlation level. The women with degenerative changes of the lumbar spine had significantly higher BMD in comparison to individuals without these changes [19]. BMD showed increased sensitivity for osteoporosis diagnosis when BMD is measured at multiple sites with the lowest T-score [22–24].

Prior studies were conducted to determine the threshold of HU on CT for the diagnosis of osteoporosis. One of the studies with a large cohort performed by Pickhardt et al., which established an optimal threshold of 135 HU at L1 with an AUROC of 0.83, and reported that a threshold of 110 HU showed more than 90% specificity [25]. Other studies demonstrated that 99 HU [26] and 90 HU [16] were optimal thresholds for diagnosing osteoporosis. It would be reasonable to assume that a lower HU threshold would show higher specificity and lower sensitivity for diagnosing osteoporosis. On the other hand, a higher HU threshold would show higher sensitivity and lower specificity. Graffy et al reported that at an optimal threshold of L1 attenuation at 90 HU, the sensitivity was 86.9% and the specificity was 83.9%, and it could also determine the risk for osteoporotic vertebral fracture [16]. Furthermore, L1 attenuation ≤90 HU was significantly associated with decreased fracture-free survival (*P* < .001) [11].

The present study has several limitations. First, it was a retrospective study, and thus has an inherent selection bias. Of note, some of the patients were not included because they had not been monitored for more than 1 year, which may have led to a greater selection bias. Second, our study was performed in a single clinical institution for Asian women with a small study size. Further studies in patients with breast cancer that includes various races. Third, because only a single reviewer measured the attenuation value once, we could not test the intra-observer agreement. The non-automated nature of attenuation measurement could make the attenuation value more variable. Fourth, we did not assess the dose or duration of steroids or aromatase inhibitors, or the extent of smoking. It is necessary to be cautious when interpreting these factors in this study. Finally, the attenuation value was only measured at a single axial scan of L1, so it may not represent the bone quality of the entire vertebra.

In conclusion, L1 attenuation ≤ 90 HU may be used to discriminate assessment risk for incidental fracture in patients with breast cancer. Chest CT scan could increase opportunities to evaluate osteoporosis and discriminate incidental fracture risk.

## Supporting information

S1 Appendix STROBE Statement—Checklist of items that should be included in reports of observational studies. (DOC)

S1 Fig a-c. Raw data of Fig 4. (TIF)

S1 Table Details in Table 3. (DOCX)

## Abbreviations

AUROC: Area under the receiver operating curve
BMD: Bone mineral density
CI: Confidence interval
CT: Computed tomography
DXA: Dual-energy X-ray absorptiometry
HU: Hounsfield unit
ROI: Region of interest
