# Supplementary figures and images for: Opportunistic use of chest CT for screening osteoporosis and predicting the risk of incidental fracture in breast cancer patients: A retrospective longitudinal study
Source: PLoS One. 2020 Oct 14;15(10):e0240084. doi: 10.1371/journal.pone.0240084 (PMC7556442; doi:10.1371/journal.pone.0240084)

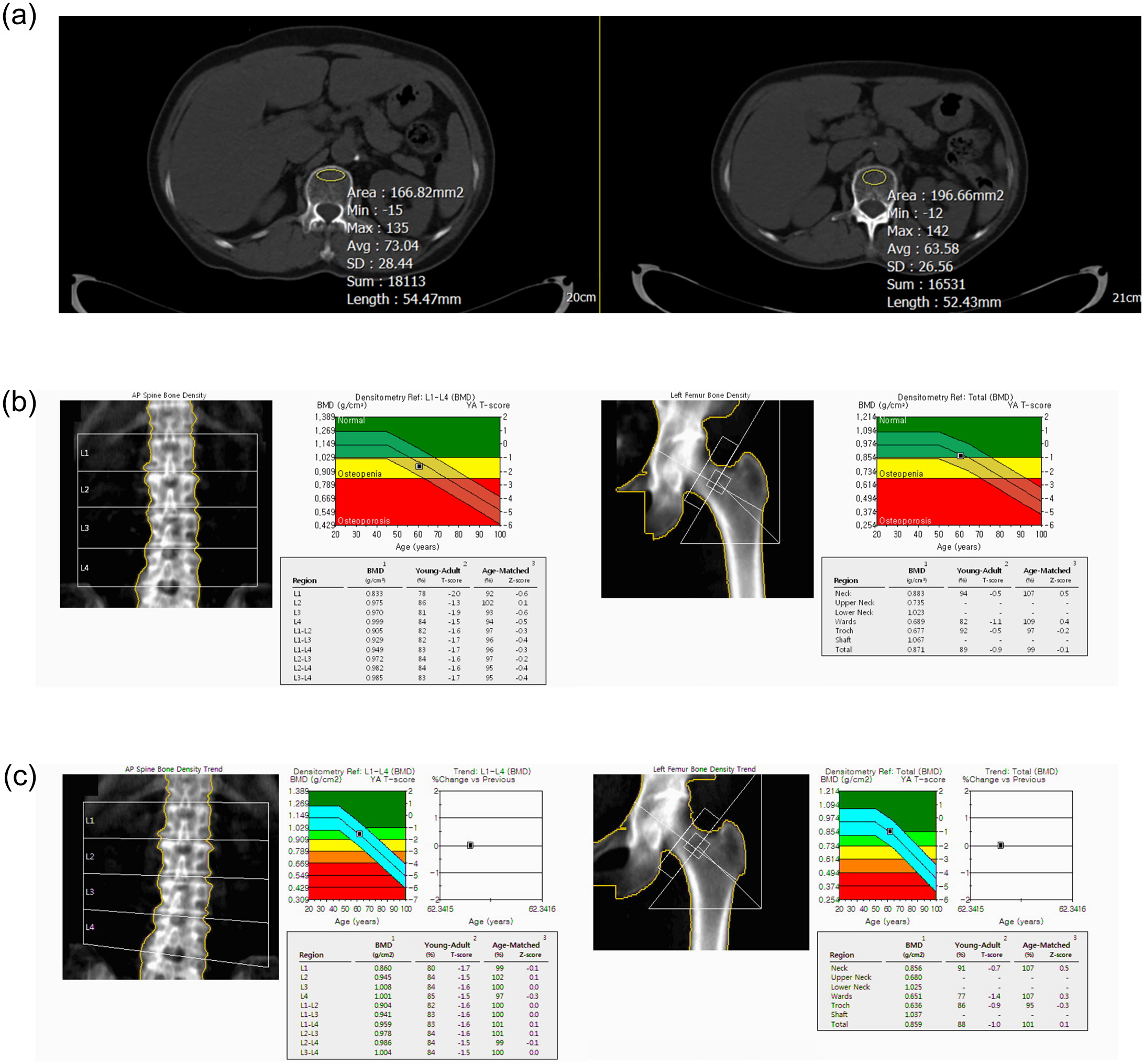

Supplement: S1 Fig — (TIF) [file pone.0240084.s002.tif]
