# Supplementary material for: Opportunistic use of chest CT for screening osteoporosis and predicting the risk of incidental fracture in breast cancer patients: A retrospective longitudinal study
Source: PLoS One. 2020 Oct 14;15(10):e0240084. doi: 10.1371/journal.pone.0240084 (PMC7556442; doi:10.1371/journal.pone.0240084)
Supplement: S1 Table — (DOCX) [file pone.0240084.s003.docx]

| Supporting information  **Diagnostic performance of L1 vertebral attenuation on chest CT for osteoporosis defined as DXA T-score ≤-2.5 (**The values behind the means, standard deviations and other measures reported) | | | |
| --- | --- | --- | --- |
|  | **Statistic** | **Value** | **95% CI** |
|  | Sensitivity | 55.01% | 49.92% to 60.03% |
|  | Specificity | 85.91% | 83.43% to 88.14% |
|  | Positive Likelihood Ratio | 3.9 | 3.24 to 4.70 |
|  | Negative Likelihood Ratio | 0.52 | 0.47 to 0.59 |
|  | Disease prevalence (*) | 30.65% | 28.13% to 33.27% |
|  | Positive Predictive Value (*) | 63.31% | 58.89% to 67.52% |
|  | Negative Predictive Value (*) | 81.20% | 79.42% to 82.87% |
|  | Accuracy (*) | 76.44% | 74.00% to 78.75% |
|  | 1. 90HU |  |  |
|  | **Statistic** | **Value** | **95% CI** |
|  | Sensitivity | 74.04% | 69.38% to 78.32% |
|  | Specificity | 78.64% | 75.78% to 81.30% |
|  | Positive Likelihood Ratio | 3.47 | 3.01 to 3.99 |
|  | Negative Likelihood Ratio | 0.33 | 0.28 to 0.39 |
|  | Disease prevalence (*) | 30.65% | 28.13% to 33.27% |
|  | Positive Predictive Value (*) | 60.50% | 57.12% to 63.79% |
|  | Negative Predictive Value (*) | 87.26% | 85.24% to 89.05% |
|  | Accuracy (*) | 77.23% | 74.82% to 79.51% |
|  | 1. 100 HU |  |  |
|  | **Statistic** | **Value** | **95% CI** |
|  | Sensitivity | 83.80% | 79.76% to 87.32% |
|  | Specificity | 69.77% | 66.62% to 72.79% |
|  | Positive Likelihood Ratio | 2.77 | 2.48 to 3.09 |
|  | Negative Likelihood Ratio | 0.23 | 0.18 to 0.29 |
|  | Disease prevalence (*) | 30.65% | 28.13% to 33.27% |
|  | Positive Predictive Value (*) | 55.07% | 52.35% to 57.76% |
|  | Negative Predictive Value (*) | 90.69% | 88.56% to 92.46% |
|  | Accuracy (*) | 74.07% | 71.57% to 76.47% |
|  | 1. 110 HU |  |  |
|  |  |  |  |
|  | **Statistic** | **Value** | **95% CI** |
|  | Sensitivity | 80.98% | 76.72% to 84.76% |
|  | Specificity | 74.09% | 71.06% to 76.96% |
|  | Positive Likelihood Ratio | 3.13 | 2.77 to 3.53 |
|  | Negative Likelihood Ratio | 0.26 | 0.21 to 0.32 |
|  | Disease prevalence (*) | 30.65% | 28.13% to 33.27% |
|  | Positive Predictive Value (*) | 58.01% | 55.02% to 60.94% |
|  | Negative Predictive Value (*) | 89.81% | 87.73% to 91.57% |
|  | Accuracy (*) | 76.20% | 73.76% to 78.52% |
|  | 1. Optimal 107 HU |  |  |
|  |  |  |  |
